# Supplementary material for: Balanced Cellular and Humoral Immune Responses Targeting Multiple Antigens in Adults Receiving a Quadrivalent Inactivated Influenza Vaccine
Source: Vaccines (Basel). 2021 Apr 23;9(5):426. doi: 10.3390/vaccines9050426 (PMC8146362; doi:10.3390/vaccines9050426)
Supplement: Supplementary file 1 [file vaccines-09-00426-s001.zip › Vaccines_Yu_Supplementary Materials Figure S1-6 and Table S5-6.pdf]

Supplementary Materials for

Balanced cellular and humoral immune responses targeting multiple antigens in adults receiving a quadrivalent inactivated influenza vaccine

Esther Dawen Yu[1], Alba Grifoni[1], Aaron Sutherland[1], Hannah Voic[1], Eric Wang[1], April Frazier[1], Natalia Jimenez-Truque[2], Sandra Yoder[2], Sabrina Welsh[3], Stacey Wooden[4], Wayne Koff [3], Buddy Creech[2], Alessandro Sette[1,5,†,*] and Ricardo da Silva Antunes[1,†,*]

[1] Center for Infectious Disease and Vaccine Research, La Jolla Institute for Immunology, La Jolla, California, USA

[2] Vanderbilt Vaccine Research Program, Vanderbilt University Medical Center, Nashville, TN, USA

[3] Human Vaccines Project, New York, NY, USA

[4] Merck & Co., Inc., Kenilworth, NJ, USA

[5] Department of Medicine, Division of Infectious Diseases and Global Public Health, University of California San Diego, La Jolla, California, USA

[†] These authors contributed equally

Conflict of interest statement: Stacey Wooden is employed and receives salaries from Merck & Co., Inc. The rest of the authors declare no conflict of interest.

[*]Correspondence should be addressed to Ricardo da Silva Antunes, La Jolla Institute for Immunology, La Jolla, CA 92037, USA, phone: +1-858-752-6500, email: rantunes@lji.org and Alessandro Sette, La Jolla Institute for Immunology, La Jolla, CA 92037, USA, phone: +1-858-752-6919, email: alex@lji.org.

Figure S1

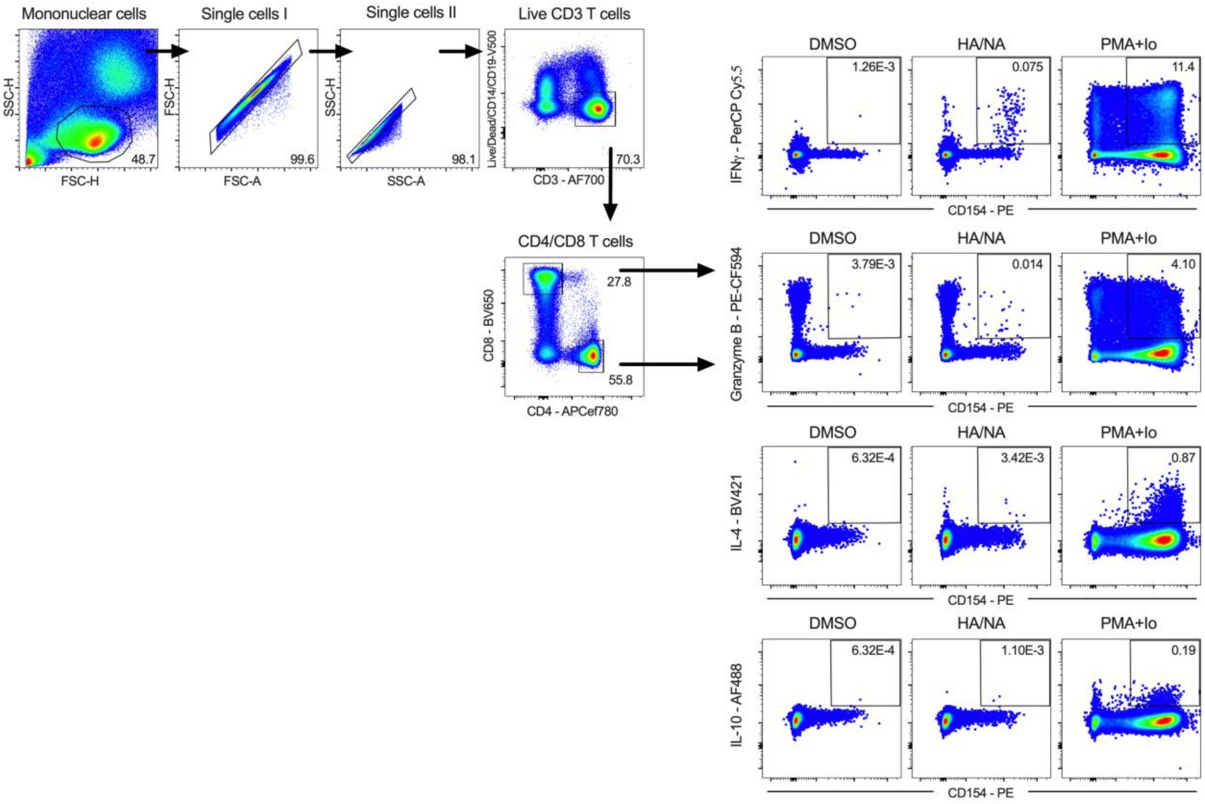

Figure S1. Gating strategy and representative cytokine responses plots of the intracellular staining (ICS) assay. Representative gating of live CD3$^+$ T cells, CD4$^+$ T cells, CD8$^+$ T cells and cytokine producing CD154$^+$ CD4 or CD8 T cells from donor PBMCs is shown. Briefly, mononuclear cells were gated out of all events followed by subsequent singlet gating. Live CD3$^+$ cells were gated as Live/Dead$^-$CD14$^-$CD19$^-$CD3$^+$. Cells were then gated as CD4+CD8$^-$ or CD4$^-$CD8+ T cells. CD4$^+$ or CD8$^+$ T cells were further subdivided into different cytokines (INFγ, Granzyme B, IL-4, IL-10) producing CD154$^+$ populations. Representative cytokines responses plots after stimulating with positive (PMA+Io) or negative (DMSO) controls and flu MegaPools were presented on the right side.

Figure S2

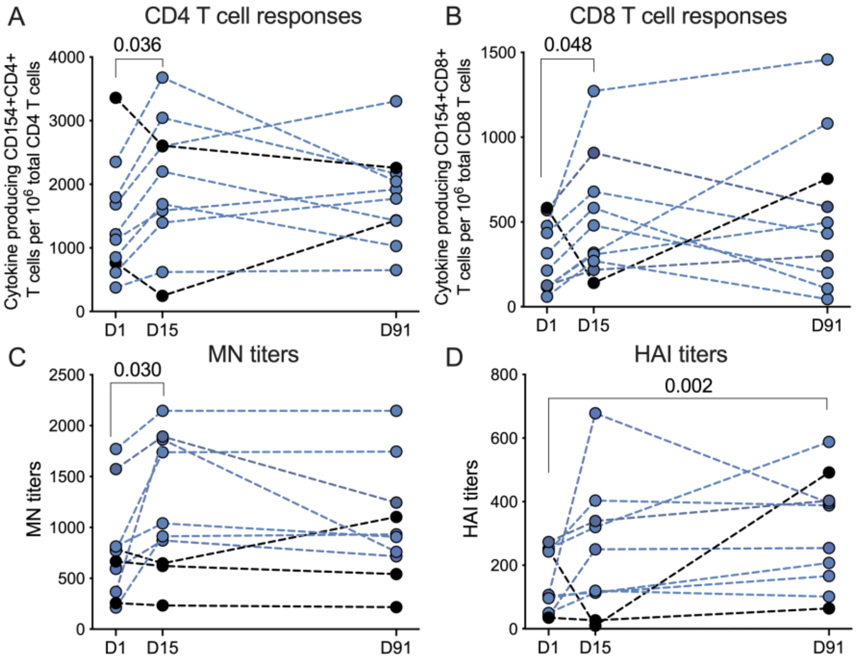

Figure S2. Individual cellular and humoral immune responses before and after vaccination. Cellular and humoral immune responses were measured at baseline (D1, pre-immunization), D15 (14 days post vaccination), and D91 (90 days post vaccination). (A, B) CD4 and CD8 T cell responses were represented by number of influenza virus reactive cytokine producing CD154+ CD4 or CD8 T cells per million of total CD4 or CD8 T cells. The individual trends of changes in CD4 and CD8 T cell responses for each visit (n=10) were shown. (C, D) Humoral immune responses were measured by both microneutralization (MN) assay and hemagglutination inhibition (HAI) assay, and total responses of all 4 strains represented. The individual trends of changes in MN or HAI antibody titers for each visit (n=10) were shown. (A-D) Normality of data distribution was accessed by Shapiro-Wilks test. Parametric data at D15 or D91 were compared to baseline (D1) by paired Student's t test (two-tailed). Non-parametric data at D15 or D91 were compared to baseline (D1) by Wilcoxon test (two-tailed). P values < 0.05 were considered statistically significant.

Figure S3

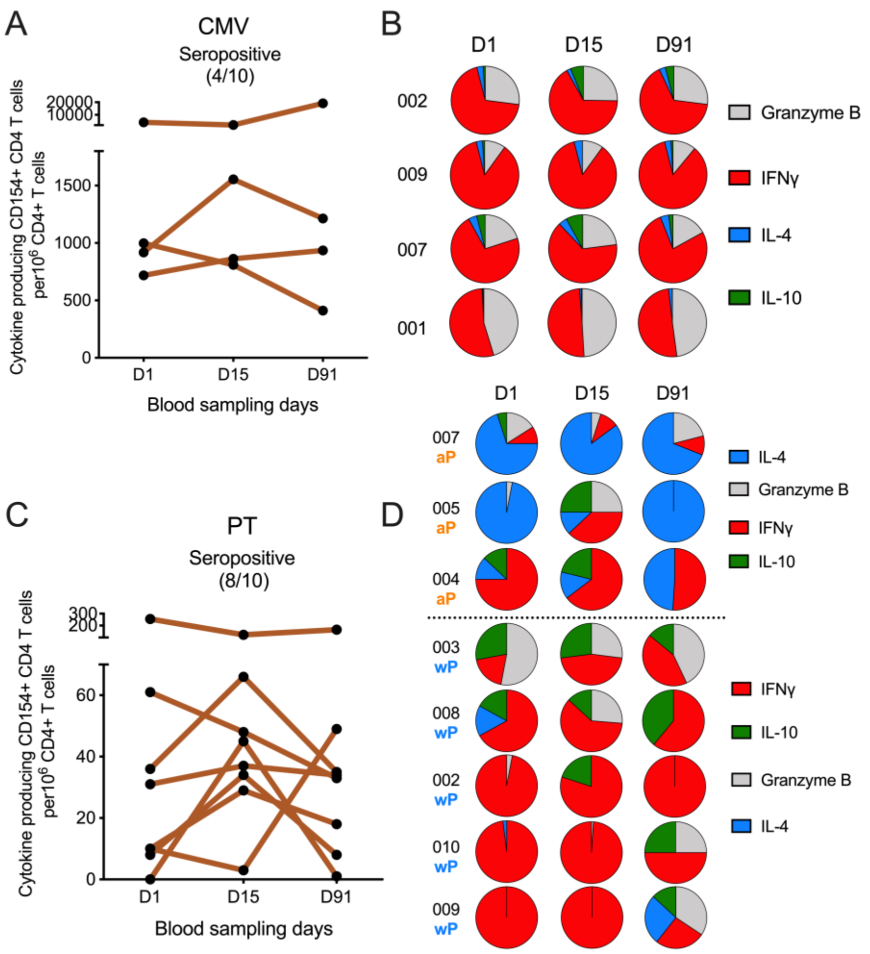

Figure S3. Cellular responses to CMV and PT control MPs do not change after influenza vaccination. Cellular responses were measured at baseline (D1, pre-immunization), D15 (14 days post vaccination), and D91 (90 days post vaccination). (**A**, **C**) The individual trends of changes in CD4 T cell responses to CMV and PT control MPs before and after vaccination were shown. (**B**) Th1 polarization shown in subjects with positive CMV response at each visit. (**D**) Th2 polarization shown in subjects with positive acellular vaccine (aP) response at each visit, and Th1 polarization in subjects with positive whole-cell vaccine (wP) response at each visit. Childhood vaccination with aP and wP were inferred by date of birth.

Figure S4

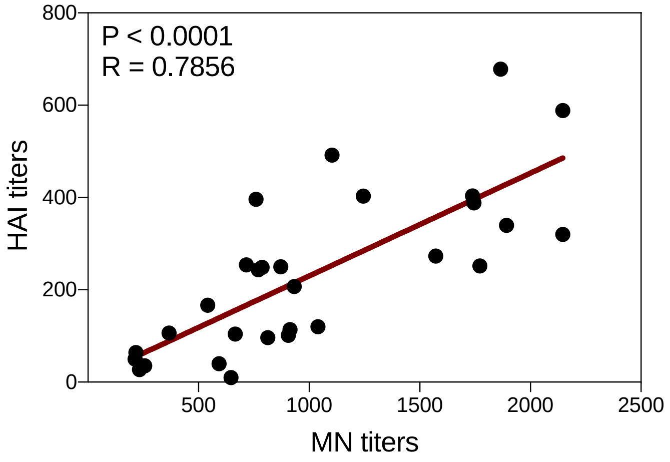

Figure S4. Total MN titers and HAI titers corelated with each other. For this analysis, we considered each time point for each individual separately. Data from all 3 visits of 10 study subjects were included and correlation were calculated by Spearman correlation test. P values < 0.05 were considered statistically significant.

Figure S5

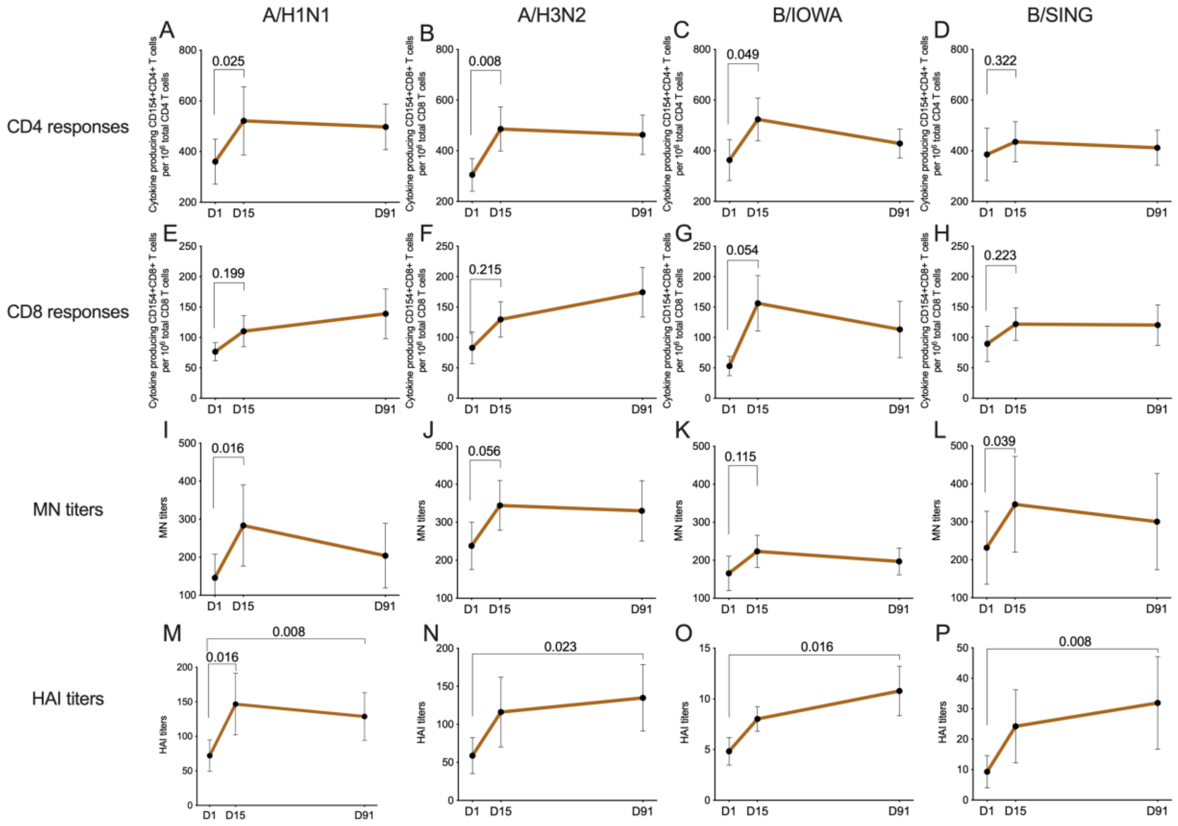

Figure S5. Longitudinal analysis of cellular and humoral immune responses after vaccination by strains. Cellular and humoral immune responses were measured at baseline (D1, pre-immunization), D15 (14 days post vaccination), and D91 (90 days post vaccination). (**A-H**) The general trends of changes in CD4 and CD8 T cell responses before and after vaccination for each influenza virus strain (A/H1N1, A/H3/N2, B/IOWA, B/SING) were shown for 10 participants at each visit. CD4 and CD8 T cell responses were represented by number of influenza viruses reactive cytokine producing $CD154^+$ CD4 or CD8 T cells per million of total CD4 or CD8 T cells. (**I-P**) Humoral immune responses were measured by both microneutralization (MN) assay and hemagglutination inhibition (HAI) assay. The general trends of changes in MN or HAI antibody titers before and after vaccination for each influenza virus strain were shown for 10 participants at each visit. (**A-P**) Normality of data distribution was accessed by Shapiro-Wilks test. Parametric data at D15 or D91 were compared to baseline (D1) by paired Student's t test (two-tailed). Non-parametric data at D15 or D91 were compared to baseline (D1) by Wilcoxon test (two-tailed). Data were plotted as median with interquartile range for non-parametric data and mean with SEM for parametric data. P values < 0.05 were considered statistically significant.

Figure S6

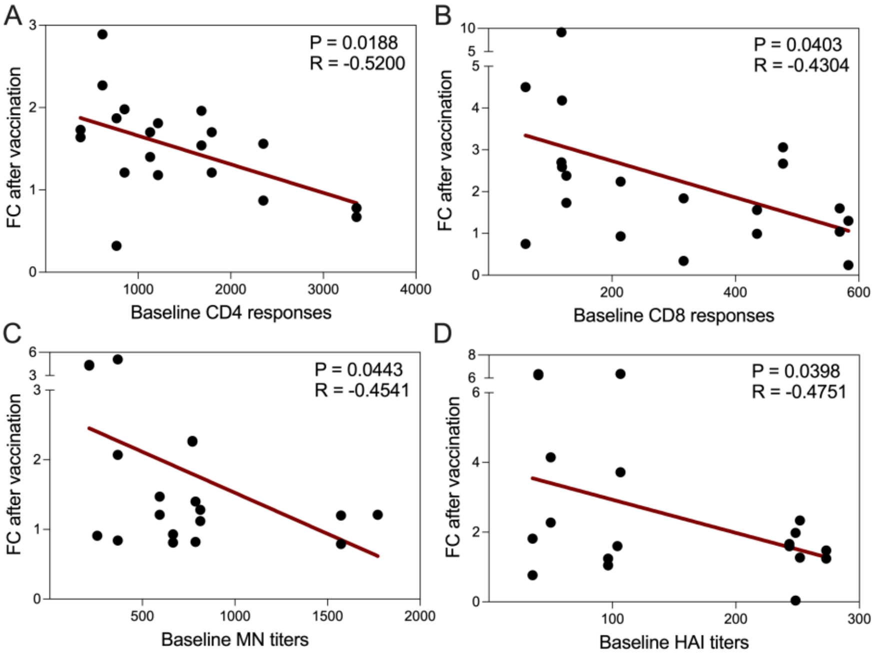

Figure S6. The "Ceiling effect" of cellular and humoral vaccine responses.

(**A** and **B**) Both baseline (D0) CD4 and CD8 responses were negatively correlated with fold of change (FC) of responses after vaccination at D14 and D90 (FC = D14 or D90/D0). CD4 and CD8 T cell responses were measured by the number of influenza viruses reactive cytokine producing CD154$^+$ CD4 or CD8 T cells per million of total CD4 or CD8 T cells. (**C** and **D**) Total antibody titers for all 4 strains of influenza viruses measured by both MN and HAI assays at baseline (D0) shown negative correlation with fold of change (FC) of antibody responses after vaccination at D14 and D90 (FC = D14 or D90/D0). (**A**-**D**) Correlations were assessed by Pearson correlation test for parametric data and Spearman correlation test for non-parametric data. P values < 0.05 were considered statistically significant.

Table S1-S4 are included in a separate excel file.

Table S1: Composition of the Human Influenza A and B viruses homologs of HA/NA epitopes.

Table S2: Composition of the Human Influenza A and B viruses homologs of PA/PB1 epitopes.

Table S3: Composition of the Human Influenza A and B viruses homologs of PB1/PB2 epitopes.

Table S4: Composition of the Human Influenza A and B viruses homologs of OTHER (NP/M1/M2/NS1/NEP) epitopes.

Table S5. List of antibodies used in the intracellular cytokine staining (ICS) assay.

Antibody	Fluorochrome	Clone	Vendor	Catalog number
CD3	AF700	UCHT1	Invitrogen	56-0038-42
CD4	APCef780	RPA-T4	Invitrogen	47-0049-42
CD8	BV650	RPA-T8	Biolegend	301042
CD14	V500	M5E2	BD Biosciences	561391
CD19	V500	HIB19	BD Biosciences	561121
CD154	PE	TRAP-1	BD Biosciences	555700
Live/Dead Viability	eF506/Aqua	-	Invitrogen	65-0866-18
IFNγ	PerCP Cy5.5	4S.B3	Invitrogen	45-7319-42
Granzyme B	PE-CF594	GB11	BD Biosciences	562462
IL-10	AF488	JES3-9D7	Invitrogen	53-7108-42
IL-4	BV421	MP4-25D2	Biolegend	500826

Table S6. Sequence similarity analysis of viral protein MPs: calculated as number (and percentage) of common peptides shared between four different strains.

**HA/NA**	A/H1N1	A/H3N2	B/IOWA	B/SING
A/H1N1	204 (100%)	0 (0%)	0 (0%)	0 (0%)
A/H3N2		204 (100%)	0 (0%)	0 (0%)
B/IOWA			207 (100%)	45 (22%)
B/SING				207 (100%)

**Others**	A/H1N1	A/H3N2	B/IOWA	B/SING
A/H1N1	232 (100%)	63 (27%)	0 (0%)	0 (0%)
A/H3N2		232 (100%)	0 (0%)	0 (0%)
B/IOWA			256 (100%)	145 (57%)
B/SING				256 (100%)

**PA/PB1**	A/H1N1	A/H3N2	B/IOWA	B/SING
A/H1N1	216 (100%)	120 (56%)	2 (1%)	2 (1%)
A/H3N2		216 (100%)	2 (1%)	2 (1%)
B/IOWA			218 (100%)	170 (78%)
B/SING				218 (100%)

**PB1/PB2**	A/H1N1	A/H3N2	B/IOWA	B/SING
A/H1N1	213 (100%)	133 (62%)	0 (0%)	0 (0%)
A/H3N2		213 (100%)	0 (0%)	0 (0%)
B/IOWA			227 (100%)	184 (81%)
B/SING				227 (100%)

HA/NA MPs include overlapping peptides spanning the hemagglutinin (HA) and neuraminidase (NA) protein sequences; PA/PB1 and PB1/PB2 MPs include overlapping peptides spanning the viral polymerases (PA, PB1 and PB2) protein sequences; and "other" MPs include overlapping peptides spanning the rest of the viral proteins sequences such as the nucleoprotein (NP), matrix protein (M1and M2), non-structural protein (NS1), and nuclear export protein (NEP).
